# Integration of the Generalist Predator *Nabis americoferus* (Hemiptera: Nabidae) in a Greenhouse Strawberry Biocontrol Program with Phytoseiid Predatory Mites and the Entomopathogenic Fungus *Beauveria bassiana*

**DOI:** 10.3390/insects15010052

**Published:** 2024-01-11

**Authors:** Taro Saito, Rosemarije Buitenhuis

**Affiliations:** Vineland Research and Innovation Centre, 4890 Victoria Avenue North, Vineland Station, ON L0R 2E0, Canada; rose.buitenhuis@vinelandresearch.com

**Keywords:** biological control, strawberry, compatibility, nabidae, predatory mite, entomopathogenic fungi, western flower thrips, two-spotted spider mites

## Abstract

**Simple Summary:**

Strawberry production has been moving into greenhouses. In addition to protecting the crop from extreme weather events, controlling the production environment allows growers to produce better quality fruits at faster harvest cycles, to optimize the water and nutrient use, and to provide supplemental lighting for out-of-season production. Western flower thrips and two-spotted spider mites cause feeding damage to leaves and flowers and reduce the yield. Biological control of these pests often includes the application of phytoseiid predatory mites and entomopathogenic fungi. A family of generalist predatory hemipteran bugs, Nabidae, are known as important generalist predators in field crops, including strawberry. Methods for the use of *Nabis americoferus* as a new biological control agent have been developed for Canadian greenhouses. In the laboratory, *N. americoferus* was compatible with predatory mites, but not with the entomopathogenic fungus *Beauveria bassiana*. In a greenhouse study, the addition of *N. americoferus* to a greenhouse strawberry biological control program based on predatory mite sachets was beneficial, potentially reducing the number of sachet applications.

**Abstract:**

In strawberry production, western flower thrips (WFT) and two-spotted spider mites (TSSM) inflict feeding damage and reduce the yield. Biological control for these pests often includes phytoseiid predatory mites and entomopathogenic fungi. The hemipteran family Nabidae have been reported as prominent predators in open-field strawberry. *Nabis americoferus* Carayon is a new biocontrol agent developed in Canada. This study examined if this species was a good candidate for integration with biological control for greenhouse strawberry production. The laboratory trials showed that *Phytoseiulus persimilis* Athias-Henriot and *Amblyseius swirskii* Athias-Henriot were compatible with *N. americoferus*, especially when alternative food was available. In contrast, the nabid was not compatible with the *Beauveria bassiana* (Balsamo) GHA strain. A greenhouse cage study was conducted to determine if it was beneficial to add *N. americoferus* to the phytoseiid-mites-based biological control program for WFT and TSSM in greenhouse strawberry. The release of *N. americoferus* on a banker plant together with the placement of sachets of *Neoseiulus cucumeris* (Oudemans) and *Neoseiulus californicus* (McGregor) was beneficial, not only potentially reducing the number of sachet applications, but also providing better pest control than phytoseiid mites alone. Neither the phytoseiids nor the *N. americoferus* numbers were significantly affected by the presence of each other.

## 1. Introduction

In recent years, strawberry production has been moving into greenhouses as protected cultivation offers several advantages over conventional field production. In addition to the obvious advantage of protecting the crop from extreme weather events, controlling the production environment allows growers to produce better quality fruits at faster harvest cycles, to optimize the water and nutrient use, and to provide supplemental lighting for out-of-season, i.e., winter, production [1]. Demand for organically grown strawberries is also on the rise, and the potential profits of greenhouse-grown organic strawberries and greenhouse-grown conventional strawberries are as much as 9.5 times and 1.5 times greater than field production, respectively [2]. 

Western flower thrips (WFT), *Frankliniella occidentalis* Pergande (Thysanoptera: Thripidae), and two-spotted spider mites (TSSM), *Tetranychus urticae* Koch (Acari: Tetranychidae), are some of the most difficult-to-control arthropod pests in greenhouse crops worldwide, due to their small size, high reproduction rate, cryptic habits, and ability to develop resistance to chemical pest-control compounds [3,4,5,6,7]. In strawberry production, both species cause feeding damage on leaves and flowers and reduce the yield [3,8,9], but WFT especially can cause damage to the fruits (bronzing, cracking, and cat-facing injuries) [10]. Tactics for the biological control of these pests often include the application of phytoseiid predatory mites or entomopathogenic fungi [5,8,9,11,12], and combinations of both have also been recommended [13,14].

Several insects from Nabidae, a predatory hemipteran family, have good potential as biological control agents due to their global distribution, natural occurrence in several crops, and wide range of pests they can attack, including prey much larger than themselves [15]. In open-field strawberries, nabids were reported to feed on green strawberry aphids, strawberry root aphids, and potato aphids in Turkey [16]; on European tarnished plant bugs in the UK [17]; on tarnished plant bugs in eastern Canada [18]; and on two-spotted spider mites in New Zealand [19]. In a previous study, three nabid species commonly occurring in southern Ontario, *Nabis americoferus* Carayon, *Nabis roseipennis* Reuter, and *Hoplistoscelis pallescens* (Reuter), were screened for their potential as new biological control agents in Canadian greenhouses by comparing life histories and examining their laboratory predation efficacy against WFT, TSSM, greenhouse whiteflies [*Trialeurodes vaporariorum* (Westwood)], and green peach aphids [*Myzus persicae* (Sulzer)]. As a result, *N. americoferus* was determined to be the most suitable predator because this species had a relatively short life cycle, and was a significantly better predator of WFT, while showing similar efficacy against the rest of the pests tested [20]. 

When scouting for natural enemies in field strawberry in southern Ontario, *N. americoferus* was observed to be one of the most encountered predators (personal observation). This study examined if this predatory bug can be integrated with, and will add value to, a common biological control program for greenhouse strawberries. *Nabis americoferus* is a generalist predator and has a large body size (10–13 mm as an adult). This, and their voracious nature, may cause adverse effects on the phytoseiid predatory mites commonly used on greenhouse strawberry, so it is important to determine the risk of intra-guild predation (IGP). IGP occurs when natural enemies share the same prey and attack each other, especially when the prey population is low, which can reduce the biological control program efficacy [21]. In addition to phytoseiid predatory mites, microbial agents such as entomopathogenic fungi are also an important tool in greenhouse strawberry pest management, so the susceptibility of *N. americoferus* to this microbial agent also needs to be clarified. 

First, this study examined the compatibility of *N. americoferus* with phytoseiid mites and a fungal bioinsecticide commonly used on Canadian greenhouse crops. Trials were carried out in the laboratory using the following species: *Phytoseiulus persimilis* Athias-Henriot, a specialist phytoseiid mite used exclusively to control TSSM on foliage; *Amblyseius swirskii* Athias-Henriot, a generalist phytoseiid mite used to manage WFT larvae and whitefly; and *Beauveria bassiana* (Balsamo) GHA strain, an entomopathogenic fungus with a wide host range. Second, a greenhouse cage study was conducted in order to determine if it is beneficial to add *N. americoferus* to the phytoseiid-mites-based biological control program for WFT and TSSM on greenhouse strawberry. The study focused on preventative biocontrol in the winter months (November–February), a period when greenhouse strawberries replace field-grown strawberries on the Canadian market. Hewitt et al. [22] compared *A. swirskii* and *Neoseiulus cucumeris* (Oudemans), another generalist phytoseiid mite used to manage WFT larvae and TSSM, for WFT control in summer and winter climates, and concluded *N. cucumeris* was a more cost-effective choice for winter months, whereas *A. swirskii* performed better in the summer and was equally good under a winter climate. *Phytoseiulus persimilis* is a TSSM specialist, and is less suited for preventative application, while *Neoseiulus californicus* (McGregor), a generalist phytoseiid mite used to manage TSSM and WFT larvae, can survive on other mites, thrips, and pollen in the absence of TSSM [23]. For those reasons, *N. cucumeris* and *N. californicus* were chosen for the greenhouse study. 

## 2. Materials and Methods

### 2.1. Nabis Americoferus Colony

A colony of *N. americoferus* was established from individuals collected in a grass field at the Vineland Research and Innovation Centre in Lincoln, ON, Canada in 2017. In later years, the colony was periodically supplemented with freshly caught individuals from the same location. Close-aged adult cohorts were reared as described in Saito et al. [20]. The current study released approximately 50 females and 5 males in a rearing cage to start a cohort. All *N. americoferus* adults used for testing were between 7 and 10 days old, after which they were assumed to have mated and have mature eggs. In all laboratory compatibility trials, each female was isolated in an individual polystyrene vial (Falcon™ 14 mL, 17 mm diam. Ø, BD Biosciences, Franklin Lakes, NJ, USA) containing a strip of paper and stoppered with a moist cotton plug for a pre-trial starvation period of 24 h prior to testing. 

### 2.2. Compatibility Trial with Phytoseiid Mites

Laboratory trials were set up using one *N. americoferus* female versus multiple adult female phytoseiid mites to mimic the proportions of predators that are commonly encountered in a crop. *Phytoseiulus persimilis* were obtained from Koppert Canada Ltd. (Scarborough, ON, Canada) in a 100 mL bottle, containing a mixture of ca. 2000 adults and nymphs. Only adult female *P. persimilis* were used in this study. The bottle was stored at 10 °C for two days until used in the experiment. The TSSM were reared continuously in a growth chamber (16 L:8 D, 27 ± 1 °C, 60% RH) with bean plants (var. California Red Kidney, Stokes Seeds Canada Ltd., Thorold, ON, Canada). Adult female TSSM used for testing were removed directly from the colony.

Small plastic cups (opening 6 cm Ø, bottom 4 cm Ø, height 3 cm, volume 2 oz, Solo^®^ cup B200, Dart Container Corporation, Mason, MI, USA) with a vent hole in the lid (2 cm Ø, covered with a thrips-proof mesh screen) were used in the trials. Detached tomato leaflets (var. Komeett, Stokes Seeds Canada Ltd.) were collected, and the petioles were dipped in 2% agar (cooled to approx. 40 °C) to prolong the freshness of the leaflets. Each cup contained a piece of wood wool (undyed Aspen fine excelsior, Uline Canada, Milton, ON, Canada) folded loosely, and the tomato leaflet was placed on the wood wool so that the arthropods could access both adaxial and abaxial sides of the leaflet. This setup provided more surface area and hiding places for the arthropods in the cup. *Phytoseiulus persimilis* were collected and transferred from the original bottles using a fine-tip paintbrush (size 000), and a pair of featherweight forceps were used to collect and transfer *N. americoferus* adult females (7–10 days old). There were two food items used, both of which were provided in excess: 35 TSSM adult females for *P. persimilis* and *N. americoferus*, and/or a frozen *E. kuehniella* egg strip (0.5 × 1.5 cm or coated with ca. 500 eggs) for *N. americoferus*. After the predators were placed inside the test cups, the snap-on lid was tightly placed. Six treatments were tested: 1. thirty-five adult female TSSM, no predators; 2. thirty-five adult female TSSM and two *P. persimilis*; 3. thirty-five adult female TSSM and one *N. americoferus* adult female; 4. thirty-five adult female TSSM, one *E. kuehniella* egg strip, and one *N. americoferus*; 5. thirty-five adult female TSSM, two *P. persimilis*, and one *N. americoferus*; and 6. thirty-five adult female TSSM, one *E. kuehniella* egg strip, two *P. persimilis*, and one *N. americoferus*. Water was not provided. The test containers were held in a growth chamber (16 L:8 D h, 25 ± 1 °C, 60% RH). The number of live and dead individuals was recorded 24 h later for all the species, as well as the number of *P. persimilis* eggs (laid on the tomato leaflet). Although the number of *N. americoferus* eggs (laid into the petioles and main leaf vain) was recorded, these data were omitted due to the inconsistency of oviposition occurring in the 24 h period. The first trial (*n* = 6 per treatment) was set up three days after receipt of *P. persimilis*. The whole trial was repeated once more using freshly ordered *P. persimilis*, yielding a total of *n* = 12 per treatment. 

The number of dead TSSM (all treatments), dead *P. persimilis* (only treatments 2, 5, and 6), and *P. persimilis* eggs (only treatments 2, 5, and 6) was analyzed by a generalized linear mixed model using Proc GLIMMIX (α = 0.05), and Gaussian distribution was assumed (α = 0.05, SAS Studio, SAS Institute Inc., Cary, NC, USA) with the treatments as a fixed factor, and the trial repetition block as a random factor. A Tukey–Kramer multiple comparison was used to contrast the results.

*Amblyseius swirskii* were purchased from Koppert Canada Ltd. (Scarborough, ON, Canada) in mini sachets (Swirski-Mite Plus, 250 mites per sachet). When they arrived, the contents of three sachets were transferred into a 500 mL disposable plastic food container cup with a ventilation hole (6 cm Ø) in the lid covered with a thrips-proof screen, and it was stored at 15 °C to let the predatory mite population mature. Only female adults were used in this study. Frozen *E. kuehniella* egg strips (0.5 × 3.0 cm or ca. 1000 eggs) were used as food for both *A. swirskii* and *N. americoferus*. Three treatments were tested: 1. one egg strip and five *A. swirskii*; 2. one egg strip and one *N. americoferus*; and 3. one egg strip, five *A. swirskii*, and one *N. americoferus*. Water was not provided. The first trial (*n* = 10 per treatment) was set up two days after opening the sachet, and the whole trial was repeated two days later, yielding a total of *n* = 20 per treatment. The number of dead *A. swirskii* and *A. swirskii* eggs was analyzed in the same manner as in the *P. persimilis* trial.

### 2.3. Compatibility Trial with Beauveria Bassiana

The susceptibility of *N. americoferus* to *B. bassiana* was assessed in a laboratory bioassay, using BotaniGard^®^ 22WP (wettable powder formulation of GHA strain, Certis Biologicals, MD, USA). The viability of *B. bassiana* conidia was determined prior to the assays as described in Saito and Brownbridge [13] and used to adjust the dilution ratio to achieve the target test concentrations. The mean viability was 85% for the first trial and 82% for the second trial. Two test concentrations were prepared in deionized water for the bioassays: 1 × 10^5^ and 1 × 10^7^ viable conidia/mL, which are both lower than the recommended rate on the BotaniGard^®^ 22WP product label for WFT control (5.5 × 10^7^–1.1 × 10^8^ conidia/mL). The exposure arena consisted of a sterile tight-fit Petri dish (50 mm Ø, PALL Corporation, MI, USA) lined with a sterilized filter paper (Whatman™, #1, 55 mm Ø, Cytiva, Marlborough, MA, USA). The lid had a screened vent hole (20 mm Ø) and also an access hole (8 mm Ø, plugged with a foam ear plug) for introducing insects into the dish. Three treatments were tested: deionized water only as the control, *B. bassiana* low rate 1 × 10^5^ conidia/mL, and *B. bassiana* high rate 1 × 10^7^ conidia/mL. Twenty dishes were prepared for each treatment (*n* = 20 per treatment, 10 males and 10 females). First, the filter paper was inoculated with 0.3 mL of the assigned fungal or control treatment; one *N. americoferus* was then placed in a dish via the hole in the lid and was provided with frozen *E. kuehniella* eggs (0.02 g per dish) scattered on the filter paper as food. Dishes were held in a growth chamber (16 L:8 D h, 25 ± 1 °C, 60% RH) for 48 h. After the exposure period had elapsed, using a pair of featherweight forceps (sterilized with 70% ethanol for each transfer), each surviving bug was transferred into an individual small plastic cup (see Section 2.1), containing a piece of fresh organically grown French bean (purchased in a grocery store, triple water-rinsed, as a water source and oviposition substrate) and an *E. kuehniella* egg strip (0.5 × 1.5 cm), allowing the bug to feed ad libitum. Survival and oviposition were assessed every 48 h for a further 12 days (14 days total observation period); a fresh bean piece and an egg strip were provided every 48 h. Dead individuals were placed on a glass slide in a Petri dish lined with a moist filter paper and incubated at 25 °C to promote outgrowth of fungi to confirm death by mycosis. 

The whole trial was repeated once more, using a different mass-reared cohort, staggered seven days after the start of the first trial. The mortality data were analyzed with Fisher’s exact test (*n* = 40, α = 0.05, Proc FREQ). The odds ratios were used to contrast the results. The differences in the mortality between sexes were also compared within the same treatment using Fisher’s exact test (*n* = 20 per sex). To see the potential effect of the fungus on the fitness of the female *N. americoferus*, the eggs observed in every 48 h period as well as the total number of eggs observed per female from each treatment (*n* = 20) were analyzed by a generalized linear mixed model using Proc GLIMMIX (α = 0.05) with the treatments as a fixed factor, a Poisson distribution, and log link. Tukey’s multiple comparison was used to contrast the results. 

### 2.4. Greenhouse Strawberry Trial

Greenhouse strawberry plugs (var. Albion) were purchased on 5 October 2022. They were transplanted into 4 L pots with peat-based standard growing media (one plant per pot). Additionally, one 3 L pot of banker plants (barley) was seeded in each of the cages preassigned for *N. americoferus* treatments, on which bird-cherry oat aphids were inoculated as soon as the seedlings emerged. There were 24 walk-in dome-shaped cages (160 cm × 160 cm × 180 cm, BugDorm©, MegaView Science, Taichung, Taiwan) placed in four rows of six cages in a greenhouse compartment. The greenhouse conditions were set to 24–26 °C daytime, 20–22 °C nighttime, and supplemental HPS lights were used to maintain a photoperiod of 16:8 h light:dark. These settings ensured that venting and cooling occurred on sunny days and additional heating was applied at night during the Canadian winter. Using a randomized complete block design, each cage containing six potted strawberries elevated on plant pot risers represented one replicate. 

The plants were left to grow for one week, and then all the predators were released preventatively, one week prior to the pest release (−1 week). The mite sachets were slow-release breeding sachets containing feeder mites for the predatory mites, and *N. americoferus* had the aphid banker plant to keep them fed and allow them to start reproducing. A pre-treatment count of predators was conducted just before the pest release at week 0. Pests were released in two inundative waves: the first release was at week 0 at the rate of 35 WFT (30 females, 5 males) and 30 TSSM females; the second release took place immediately after week 5 data were taken, at the rate of 70 WFT (60 females, 10 males) and 30 TSSM females. Arthropod populations (adult and juvenile stages combined) were visually assessed weekly up to 12 weeks on all six strawberry plants in each cage. 

Treatments were:

1. The phytoseiid full recommended rate: one *N. cucumeris* sachet (Thripex-plus, 1000 mites per sachet, Koppert Canada Ltd.) and one *N. californicus* sachet (Spical-plus, 100 mites per sachet, Koppert Canada Ltd.) per cage at −1 week; repeated at 6 weeks, just after the second wave of pests was released.
2. The phytoseiid full rate plus *N. americoferus*—same as treatment 1, plus two female and two male *N. americoferus* at −1 week.
3. The phytoseiid half rate plus *N. americoferus*—initial treatment the same as treatment 2, but the predatory mite sachets were NOT repeated.

The observed numbers of arthropods from each cage (*n* = 8 cages per treatment, WFT, TSSM, phytoseiid mites, and *N. americoferus*) were analyzed with a generalized linear mixed model using Proc GLIMMIX (α = 0.05, SAS Studio, SAS Institute Inc., Cary, NC, USA) with repeated measures. The treatment and time, and their interaction were considered as fixed factors. An AR(1) covariance structure was also chosen to consider the dependency between observations taken over time on the same cage. Separate models were created for each arthropod category. A least-squares means test was used to evaluate the significance of main effects. A Tukey–Kramer multiple comparison was used to evaluate post-hoc differences among the treatments in each sampling week. 

## 3. Results

### 3.1. Compatibility Trial with Phytoseiid Mites

There were significant differences when the number of dead TSSM was compared across the treatments, showing the relative efficacy of *P. persimilis* and *N. americoferus* against this pest, as well as the effect of the presence of *E. kuehniella* eggs on the efficacy of *N. americoferus* on TSSM (F _5, 65_ = 29.06, *p* < 0.001, Table 1). One *N. americoferus* female ate the highest number of TSSM, almost the double of what two *P. persimilis* females ate together, and combining the two predator species was not better than *N. americoferus* alone. Having access to *E. kuehniella* eggs significantly reduced the predation of *N. americoferus* on TSSM. The corrected mortality values show the relative efficacy of all treatments killing TSSM (Table 1). 

There was indeed IGP observed (F _2, 32_ = 4.92, *p* = 0.014, Table 1), and the number of dead *P. persimilis* was significantly higher when confined with *N. americoferus* compared with *P. persimilis* alone. However, having access to *E. kuehniella* eggs not only significantly reduced the efficacy of *N. americoferus* killing TSSM, but it also significantly reduced the mortality of *P. persimilis,* leading to a corrected mortality value of 0% (Table 1). Most of the *P. persimilis* laid eggs, and the number of eggs was not significantly different (F _2, 32_ = 1.49, *p* = 0.24, Table 1) when the mites were alone compared to when they were with *N. americoferus*. All *N. americoferus* survived the trial, which means that the IGP relationship between the adult stages of *N. americoferus* and *P. persimilis* is unidirectional (the intra-guild predator *N. americoferus* > the intra-guild prey *P. persimilis*). 

No *N. americoferus* died while confined with five *A. swirskii* for 24 h. In contrast, 1.8 out of 5 *A. swirskii* were eaten by *N. americoferus*, which was significantly more than the 0.75 dead *A. swirskii* in the conspecific-only treatment (F _2, 37_ = 8.56, Table 2). The corrected percent mortality due to IGP showed a similar value (24.71%) to that of *P. persimilis* without an alternative food source (26.04%, Table 1). The number of eggs laid by *A. swirskii* was not significantly different (F _2, 32_ = 1.49, Table 2) when the mites were alone compared to when they were with *N. americoferus*. The IGP relationship between the adult stages of *N. americoferus* and *A. swirskii* is unidirectional (the intra-guild predator *N. americoferus* > the intra-guild prey *A. swirskii*). 

### 3.2. Compatibility Trial with Beauveria Bassiana

The mortality of *N. americoferus* was significantly higher in the treatment with the high fungal concentration, whereas the low concentration did not cause a significant increase in *N. americoferus*’s mortality compared to the untreated control treatment (Table 3). Fungal growth (mycosis) was observed on 26 isolated cadavers out of 29 dead (65.0% mortality due to mycosis) in the high-concentration treatment, and on 10 out of 12 dead (25.0% mortality due to mycosis) in the low-concentration treatment. There was no statistical difference in mortality between the females and males when compared within the same treatment (untreated control *p* = 0.16, low concentration *p* = 0.22, and high concentration *p* = 0.16).

The oviposition rate over 48 h per female differed significantly among the treatments (F _2, 57_ = 3.76, Table 3), where the *N. americoferus* females exposed to the high *B. bassiana* concentration laid significantly more eggs than those in the control. The females exposed to the low concentration laid more eggs numerically than those in the control, but this was not statistically significant. The total number of eggs laid per female during the observation period also differed significantly among the treatments (F _2, 57_ = 25.53, Table 3). The *N. americoferus* females exposed to the high *B. bassiana* concentration laid significantly fewer eggs than those in the control and low-concentration treatments, owing to the fact that the high concentration killed more than half of the females by the eighth day of the observation period. Contrastingly, the exposure to the low concentration significantly increased the oviposition during the observation period, which is because the females in the low fungal concentration survived relatively well and had a numerically higher oviposition rate than the ones in the control.

### 3.3. Greenhouse Strawberry Trial

**Figure 1:**
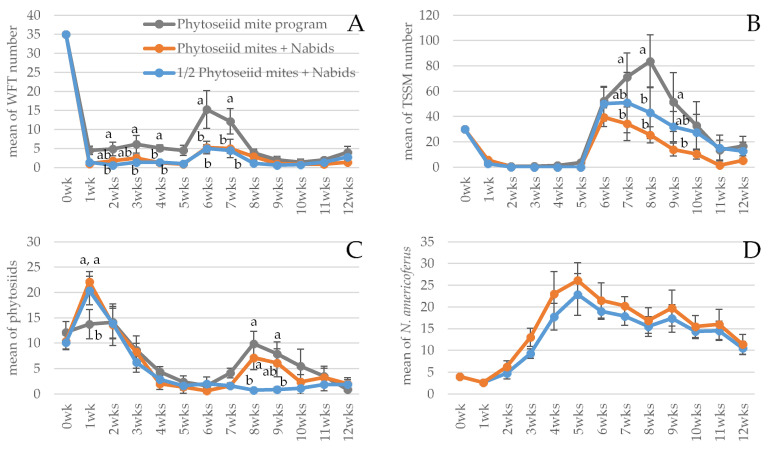
Mean (±SE) arthropod numbers over time per cage (Y-axis, six potted strawberries per cage, one plant per pot) under three biological control treatments. Note that the phytoseiid mites were released at −1 week (wk), and 6 weeks (wks) for phytoseiid mites full rate treatments; *N. americoferous* at −1 week; and the pests were released at 0 week and after 5 weeks. (**A**) Western flower thrips; (**B**) two-spotted spider mite; (**C**) phytoseiid mites, *Neoseiulus cucumeris*, and *N. californicus* combined; and (**D**) *Nabis americoferus*. Means within a panel and within a sampling period marked with different letters are significantly different (Tukey’s test: *p* < 0.05).

Both the treatments and the time had an effect on WFT numbers, and there was no interaction of these factors (treatment: F _2, 21_ = 19.17, *p* < 0.001; time: F _12, 252_ = 146.89, *p* < 0.001; and treatment × time: F _24, 252_ = 1.46, *p* = 0.08). The treatment effects were significantly higher for the two treatments where *N. americoferus* was added compared to the biocontrol program based entirely on phytoseiid predator sachets. When the population was assessed among the treatments within a sampling time point, there were significant differences among treatments at 2 weeks, 3 weeks, 4 weeks, 6 weeks, and 7 weeks (Figure 1A). Significantly higher WFT populations were observed in the phytoseiid-mites-alone treatment, compared to the other two treatments with *N. americoferus* added. There were no significant differences between the two treatments with *N. americoferus*. 

Only the factor time had a significant effect on TSSM population numbers; treatments had no effect and there was no interaction (treatment: F _2, 21_ = 1.88, *p* = 0.18; time: F _12, 252_ = 15.29, *p* < 0.001; treatment × time: F _24, 252_ = 0.95, *p* = 0.54). All the treatments suppressed the first wave of TSSM very well to a level where they were visually undetectable, but the second wave of TSSM, released after 5 weeks, persisted better than the first wave. While the phytoseiid-mites-alone treatment took two weeks to start impacting the TSSM population, the phytoseiid half rate plus *N. americoferus* treatment took only one week, and the mite full rate plus nabid treatment did not allow the population to increase at all. When the population was assessed among the treatments within each sampling time point, there were significant differences among treatments at 7 weeks, 8 weeks, and 9 weeks (Figure 1B). The number of TSSM in the phytoseiid-mite-alone treatment was significantly higher than in the phytoseiid full rate plus *N. americoferus* treatment at those time points; in contrast, the phytoseiid half rate plus *N. americoferus* treatment worked significantly better than the phytoseiid-alone treatment at 8 weeks. 

Phytoseiid mites persisted in all treatments. Only the factor time had an effect on the population numbers, but the treatment factor did not and there was no interaction (treatment: F _2, 21_ = 1.85, *p* = 0.18; time: F _12, 252_ = 18.01, *p* < 0.001; treatment × time: F _24, 252_ =1.32, *p* = 0.15). Within a sampling time point, there were significant differences among treatments at 1 week, 8 weeks, and 9 weeks (Figure 1C). Phytoseiid numbers peaked at two weeks post-release for both releases. Because the mite half rate plus *N. americoferus* treatment did not receive the second application of phytoseiid mites at 6 weeks, the number was significantly lower at 8 weeks than in the other two treatments that did have the second application. Overall, there was no evidence of a significant reduction in the phytoseiid mite population due to the presence of *N. americoferus*; therefore, the IGP between the tested phytoseiid mites and *N. americoferus* on the pest-infested strawberry crop was insignificant. This was further supported by the fact that the phytoseiids in the half rate plus nabid treatment persisted for 13 weeks. It is not clear why the number of phytoseiids was significantly lower in the phytoseiid-alone treatment, as seen at week 1, compared to the other two treatments with the nabids. 

*Nabis americoferus* was absent from the phytoseiid-alone treatment, so this treatment was not included in the data analysis of the number of *N. americoferus*. The population growth in the two treatments with the bugs added was affected only by time, not by treatment, and there was no interaction (treatment: F _1, 14_ = 1.42, *p* = 0.25; time: F _12, 168_ = 8.53, *p* < 0.001; time × treatment: F _12, 168_ = 0.13, *p* = 1.0). There were no significant differences at any of the time points (Figure 1D). The nymphs began appearing as early as 1 week and peaked at 5 weeks (not shown). The second generation started to mature at 4 weeks, and they were all matured by 9 weeks as the third-generation nymphs also started to appear. 

## 4. Discussion

The laboratory trials examined the compatibility of *N. americoferus* with phytoseiid mites and a fungal bioinsecticide commonly used in Canadian greenhouse crops: *P. persimilis*, a TSSM specialist phytoseiid; *A. swirskii*, a generalist phytoseiid for managing WFT larvae and whitefly; and *B. bassiana* GHA strain, an entomopathogenic fungus with a wide host range. A greenhouse cage study was then conducted during the winter months, to showcase a scenario of out-of-season strawberry production. Although it would have been more straightforward to test the same species throughout this study, alternative phytoseiid species were used in the greenhouse study in order to reflect current recommendations for preventative biocontrol in winter: i.e., *N. cucumeris*, a more cost-effective alternative to *A. swirskii*; and *N. californicus*, a generalist alternative to *P. persimilis*.

*Nabis americoferus* is about 100 times larger than phytoseiid mites, which clearly makes it an intra-guild predator. Indeed, both phytoseiid mite species suffered unidirectional IGP in a small container. Despite this, the corrected mortality of *P. persimilis* and *A. swirskii* was only ca. 25%, which is a good indication that the IGP in a real crop setting could be minimal as it is often the case that IGP is worse in laboratory settings than it would be in greenhouse environments with a real crop. 

The *P. persimilis* trial also showed that *N. americoferus* is 4× more efficacious per individual than *P. persimilis*. One *P. persimilis* adult female consumed about 4.5 TSSM in 24 h, which was similar to 3.2 TSSM in 24 h reported from another laboratory study [25]. The slightly better efficacy found in our study may be due to environmental differences between the trials such as temperature. *Nabis americoferus* performed better in eating TSSM (17.5 TSSM in this study) than they did in our previous study (8.5 TSSM) [20]. The current study employed an arena with three-dimensional surfaces, while the previous study used a two-dimensional test arena. The more complex environment may have facilitated *N. americoferus*’s hunting of TSSM in this study. However, having access to *E. kuehniella* eggs reduced their predation efficacy on TSSM by half. *Nabis americoferus* did eat most of the *E. kuehniella* eggs presented, which suggests that when *N. americoferus* find a patch of prey (‘egg strips’), they tend to stay in the prey patch until the patch is depleted, and may prefer *E. kuehniella* eggs over TSSM. 

The corrected mortality of *P. persimilis* due to IGP was reduced to nil when *N. americoferus* had access to *E. kuehniella* eggs. The nabids were most likely satiated enough to not cause an increase in the mortality of *P. persimilis*. Because the *A. swirskii* IGP trial did not have alternative food sources, it is possible that the mortality of *A. swirskii* could be reduced if another food source such as live WFT larvae was also available. Our findings were similar to those of Cloutier and Johnson [26], who subjected another generalist hemipteran predator, *Orius tristicolor* (White), to a similar test using WFT, TSSM, and *P. persimilis*. *Orius tristicolor* did feed on both WFT and *P. persimilis*, but the mortality was reduced when TSSM were also available as alternative food. The authors noted it seemed that any phytoseiids present in the microhabitat where *O. tristicolor* were searching for normal prey would be readily attacked. Chow et al. [27] tested the compatibility of a generalist hemipteran predator, *Orius insidiosus* (Say), with *A. swirskii*. They found *O. insidiosus* had no preference for *A. swirskii* over WFT, but *O. insidiosus* always switched to the more abundant prey. Similarly, our study also found no obvious preference for any of the biocontrol agents over the targeted pests and/or alternative food; actually, it seemed *N. americoferus* readily attacked any arthropods in the vicinity of where it was searching for food. This generalist character of *N. americoferus* could be a nuisance in situations where multiple agents are needed for multiple pests, but a theoretical work by Ikegawa et al. [28] showed that, even when IGP by one of the natural enemies is severe, it may still be beneficial to use multiple natural enemies if the generalist switches the main prey, depending on the relative abundance of prey species. Cloutier and Johnson [26] suggested that if the generalist predator attacking the specialist predator was dependent on prey density, increasing predation by a generalist on a specialist could possibly prevent a population crash of the specialist toward the end of a prey infestation. 

There is one stage that phytoseiid mites could attack in the life cycle of *N. americoferus*, which is the egg stage. Vangansbeke et al. [29] looked at the possibility of phytoseiids feeding on the eggs of hemipteran predators inserted inside plant tissues and concluded the phytoseiids did not affect the egg-hatching rate of anthocorid and mirid predators. This is probably the case for *N. americoferus* eggs as well, which are inserted into plant tissues but are also larger than the anthocorid and mirid eggs.

According to the classification system for side-effects of pesticides on natural enemies developed by the International Organisation for Biological Control [30], the *B. bassiana* GHA strain in BotaniGard 22WP may be considered ‘harmless to slightly harmful’ to *N. americoferus* in our low concentration as the pathogen caused 9.68% mortality, and ‘moderately harmful’ in our high concentration as the pathogen caused 64.5% mortality. However, the number of confirmed mycosis cases clearly shows that *N. americoferus* is susceptible to this pathogen. Although few other laboratory studies have looked at the compatibility of hemipteran predators and *B. bassiana*, a similar laboratory dish assay confirmed that *O. insidiosus* was susceptible to the *B. bassiana* GHA strain (BotaniGard ES) with similar mortality rates to those observed in our study [31]. There are numerous studies reporting hemipteran pests being susceptible to the *B. bassiana* GHA strain: for example, *Lygus hesperus* Knight [32], *Lygus lineolaris* (Palisot de Beauvois) [33], *Nasonovia ribisnigri* (Mosley) [34], *Lycorma delicatula* (White) [35], *Nezara viridula* (L.) [36], *Piezodorus guildinii* (Westwood) [37], and *Halyomorpha halys* (Stål) [38]. Contrastingly, the oviposition data showed that exposure to *B. bassiana* increased oviposition in *N. americoferus* females. A similar observation was made by Ramírez-Ordorica et al. [39], reporting that the volatiles from *B. bassiana* triggered female moths to lay more eggs. They suggested that the stimulation of oviposition behavior could be an ecological adaptive advantage in which the entomopathogen stimulates the insect population growth to ensure the host availability. Overall, we concluded that *N. americoferus* is susceptible to the *B. bassiana* GHA strain, and that it is not recommended to apply *B. bassiana* while *N. americoferus* is present in a crop. Therefore, *B. bassiana* was excluded from our greenhouse study. 

Prior to the strawberry trial, preliminary greenhouse trials were conducted using potted sweet peppers and tomatoes in order to determine the optimum release strategy for *N. americoferus*. The *N. americoferus* population increased the most using the aphid banker plants and the nabid was strongly affiliated to the banker plant, almost neglecting the tall crop plants. In the greenhouse trial, *N. americoferus* was observed to be affiliated with strawberry plants as strongly as with the banker plant. This was expected since the literature suggests that *N. americoferus* prefers crops that are closer to the ground such as alfalfa, soybeans, and grasses [40]. In our current study, the nabids initially reproduced in the banker plants, because the bird-cherry oat aphids were already abundant when the nabids were released, one week prior to the pest release. However, the nabids quickly spread among the strawberry plants as their population was building up. According to the raw data (not shown), the population age demographic of *N. americoferus* indicated that the second generation appeared 8–14 days post-release, took three weeks to mature, and started to decline nine weeks post-release. This aligned well with our previous data on their life history where the egg period was 8.35 days; the total nymphal period was 18.34 days; and adult longevity was 28–38.75 days [20]. The nabids are known to cannibalize, as older larger individuals readily prey on younger smaller ones [15]. The slight population decrease between 5 weeks and 6 weeks was likely due to the increasing cannibalism, because the prey population was diminishing while the *N. americoferus* population was rapidly increasing. The second generation of fresh adult nabids appeared to have quickly responded to the second wave of adult WFT as soon as the thrips were introduced after 5 weeks. They suppressed the pest population significantly better compared to the phytoseiid-mites-alone treatment, as shown by the large margin of difference at 6 weeks. The phytoseiid-alone treatment was slower to reduce the second wave of WFT, probably because phytoseiids attack mainly the first instar WFT larvae [23,41], whereas the nabid can attack all the mobile stages of WFT on the foliage, including adults [20]. The third generation of young nabid nymphs appeared after 8 weeks, and they were often observed walking on the webbing produced by TSSM and eating TSSM. The continuous presence of *N. americoferus* compared to the population fluctuations of the phytoseiid mites likely caused the large difference compared to the phytoseiid-alone treatment in the TSSM population at 8 weeks. The pest populations in the phytoseiid-alone treatment started to decrease at 8 weeks when the phytoseiid population on the foliage peaked as a result of the second application. The strawberry fruit yield data were omitted because mice were causing feeding damage periodically. However, some observations could be made: the nabids were often observed patrolling on the flowers and fruits, but no obvious deformations were observed, and only one nabid egg was found inserted into a fruit.

Although all biocontrol treatments brought both WFT and TSSM under control, the best pest control was achieved by the combination of the repeated phytoseiid mite sachets application plus *N. americoferus*. However, it is notable that the phytoseiid half rate plus nabid treatment provided better control of both WFT and TSSM, compared to the phytoseiid repeated application alone treatment. Neither the phytoseiids nor *N. americoferus* numbers were significantly affected by the presence of each other, indicating that they are functionally compatible. Combined with our laboratory compatibility trials with other phytoseiid species, this study demonstrated that the addition of *N. americoferus* to greenhouse strawberry biological control programs based on phytoseiid mite sachets is beneficial, not only potentially reducing the number of sachet applications and the associated costs, but also providing better pest control than phytoseiid mites alone. 

## Figures and Tables

**Table 1:** Mean number ± SE of dead two-spotted spider mites (TSSM), dead *Phytoseiulus persimilis* (*P. p.*), and number of *P. persimilis* eggs, 24 h after release with *Nabis americoferus* (*N. am.*) in compatibility trials, with or without alternative food (*Ephestia kuehniella* eggs). Corrected mortality was calculated using Abbott’s formula [24].

	Pest Only	With *P. p.*	With *N. am.*	With *N. am.* and *Ephestia* Eggs	With *P. p.* and *N. am.*	With *P. p.* and *N. am.* and *Ephestia* Eggs
Dead TSSM (max 35)	1.0 ± 0.35 a	9.25 ± 1.1 b	17.58 ± 1.76 c	6.08 ± 0.72 b	17.92 ± 1.43 c	11.08 ± 1.41 b
Corrected mortality	-	24.26%	48.76%	14.94%	49.76%	29.65%
Dead *P. p.* (max 2)	n/a	0.08 ± 0.08 a	n/a	n/a	0.58 ± 0.19 b	0.08 ± 0.08 a
Corrected mortality		-			26.04%	0%
*P. p.* eggs	n/a	2.33 ± 0.26	n/a	n/a	2.0 ± 0.33	1.58 ± 0.34

Note: Assigned lower case letters next to the mortality values in each row show the result of a post-hoc test; data points with different letters indicate that values are significantly different from the others.

**Table 2:** Mean number ± SE of dead *Amblyseius swirskii* and number of *A. swirskii* eggs, 24 h after release with or without *Nabis americoferus* in IGP trials. Percent corrected mortality was calculated using Abbott’s formula [24].

	*A. swirskii* Only	With *N. americoferus*	*p*-Value
Dead *A. swirskii* (max 5)	0.75 ± 0.23	1.8 ± 0.28	0.006
Corrected mortality	-	24.71%	
*A. swirskii* eggs	2.65 ± 0.35	3.15 ± 0.47	0.4

**Table 3:** *Nabis americoferus* adult mortality, mycosis and oviposition 48 h after exposure to *Beauveria bassiana* GHA strain. Corrected mortality was calculated using Abbott’s formula [24].

	Untreated Control	*B. bassiana* Low	*B. bassiana* High	*p*-Value
Number of dead (max 40, 20 of each sex)	9 a (6 females and 3 males)	12 a (5 females and 7 males)	29 b (13 females and 16 males)	<0.001
Corrected mortality	-	9.68%	64.5%	
Confirmed mycosis	0%	25.0%	65.0%	
Number of eggs laid in 48 h per female	12.56 ± 1.76 a	15.11 ± 1.47 ab	15.66 ± 2.11 b	0.029
Total eggs laid per female (14 d)	73.8 ± 10.79 b	83.35 ± 10.0 a	65.85 ± 13.4 c	<0.001

Note: Assigned lowercase letters next to the mortality values in each row show the result of a post-hoc test; data points with different letters indicate that values are significantly different from the others.

## Data Availability

The data presented in this study are available on request from the corresponding author. The data are not publicly available due to the ownership by Vineland Research and Innovation Centre.
